# Presumptive primary intrathoracic mast cell tumours in two dogs

**DOI:** 10.1186/s12917-019-1950-5

**Published:** 2019-06-17

**Authors:** Juan Carlos Cartagena-Albertus, Antoaneta Moise, Sergio Moya-García, Nora Cámara-Fernández, Jose Alberto Montoya-Alonso

**Affiliations:** 1 Northlands Vets., 2 Northampton road, Kettering, NN15 7JU UK; 2 Vetersalud Dr. Moya, Av Joan Miró, 40, 29620 Torremolinos, Spain; 3 Internal Medicine, Faculty of Veterinary Medicine, University Institute for Biomedical and Health Research (IUIBS), University of Las Palmas de Gran Canaria, Campus Universitario Cardones de Arucas, 35413 Las Palmas de Gran Canaria, Spain

**Keywords:** Canine primary intrathoracic tumour, Intrathoracic chest wall, Intrathoracic mast cell, Lung, Mast cell tumour

## Abstract

**Background:**

Mast cell tumours are the most common cutaneous neoplasms in dogs. Other primary sites include visceral organs, such as the gastrointestinal tract, liver, or spleen**,** and the oral cavity. Frequent metastatic sites include the local lymph nodes, skin, spleen, liver and bone marrow. The thorax is rarely affected by metastatic disease and no such cases have been reported in dogs.

Mast cell tumours are usually not considered as a differential diagnosis for lung and intrathoracic chest wall masses in dogs. Chest wall tumours can be primary tumours of the ribs and sternum, an invasion of adjacent tumours into the chest wall, and metastasis from distant tumours.

**Cases presentation:**

A German Shepherd dog presented with a history of persistent cough and a large mass involving the thoracic wall and a small round pulmonary mass. The dog had a history of mammary tumours that were surgically excised. Thoracoscopy revealed a thoracic wall mass involving the internal intercostal muscle and a small mass in the left cranial lung lobe. Cytology and histopathology of the intrathoracic mass confirmed the large mass as a mast cell tumour and the small mass as a carcinoma. Cytology of the sternal lymph nodes showed no involvement. The dog received toceranib for 3 months, which failed to alleviate persistent cough. Radiology indicated that the large mass had a partial response to toceranib. The dog was euthanasied.

A Maltese dog presented with a history of chronic regurgitation and cough**,** and a large mass involving the left caudal lung lobe. Cytology and histopathology of mass confirmed a mast cell tumour. The dog received toceranib for 2 months. Radiology indicated that the large mass had no response to toceranib. The dog was euthanasied. Confirmation of lungs mast cell tumour and the absence of any other Mast cell tumour was achieved by postmortem examination.

**Conclusions:**

The cases discussed are two unusual presentations of intrathoracic mast cell tumours, in the absence of cutaneous mast cell tumours, in dogs.

## Background

Mast cell tumours (MCTs) are not usually considered a differential diagnosis for solitary intrathoracic chest wall masses in dogs. Chest wall tumours can be metastases from distant tumours, adjacent tumours invading into the chest wall or primary tumours of the ribs and sternum. Primary rib tumours are the most common tumours of the chest wall and are frequently malignant sarcomas (osteosarcoma, chondrosarcoma, fibrosarcoma and haemangiosarcoma) [1–5].

MCTs are the most common cutaneous neoplasms in dogs, accounting for 16–21% of all canine skin malignancies [3]. Other primary sites include visceral organs, such as the gastrointestinal tract, liver, or spleen [4], and the oral cavity [6]. Frequent metastatic sites for canine MCTs include the local lymph nodes, skin, spleen, liver and bone marrow. The intrathoracic chest wall or lungs are rarely affected by metastatic disease of a MCT [3], and no such cases have been reported in dogs.

Another case of a dog with a pulmonary and splenic MCT had been described without cutaneous involvement [7]. Primary splenic and intestinal MCTs without pre-existing primary cutaneous involvement are more frequent in cats [8].

We found no reference to canine primary intrathoracic MCTs. In all reported cases of intrathoracic MCT, the mast cell disease always spreads to extrathoracic organs [1, 4, 6, 7, 9]. To the best of our knowledge and based on the presentation of a large solitary intrathoracic chest wall lesion and a pulmonary mass and the absence of previous cutaneous MCT, these cases study represent the first reported instances of canine presumptive primary MCTs involving the intrathoracic chest wall and the lungs.

## Case presentation 1

The Case 1 is a 9- year- old, 34.1- kg (body condition score 6/9), female, neutered German Shepherd presented with lethargy, weight loss, exercise intolerance and mild dyspnoea. The medical history of the dog included a mammary carcinoma 19 months earlier, with complete clinical resolution following mastectomy. Upon presentation, the dog was hyperthermic (39.2 °C), apathetic and approximately 5% dehydrated. She was tachycardic (150 beats/min) but had a normal auscultation, a soft abdominal palpation and no palpable external masses.

Blood analysis and supportive therapy were initially performed. All results were within reference limits.

The dog received intravenous crystalloid fluid therapy and was medicated with amoxicillin and clavulanic acid (8.75 mg/kg, SC, once daily) and ranitidine (2 mg/kg, IV, twice daily).

Occasional episodes of productive cough and vomiting were observed during hospitalisation. The dog also received maropitant (1 mg/kg, SC, once daily).

**Fig. 1:**
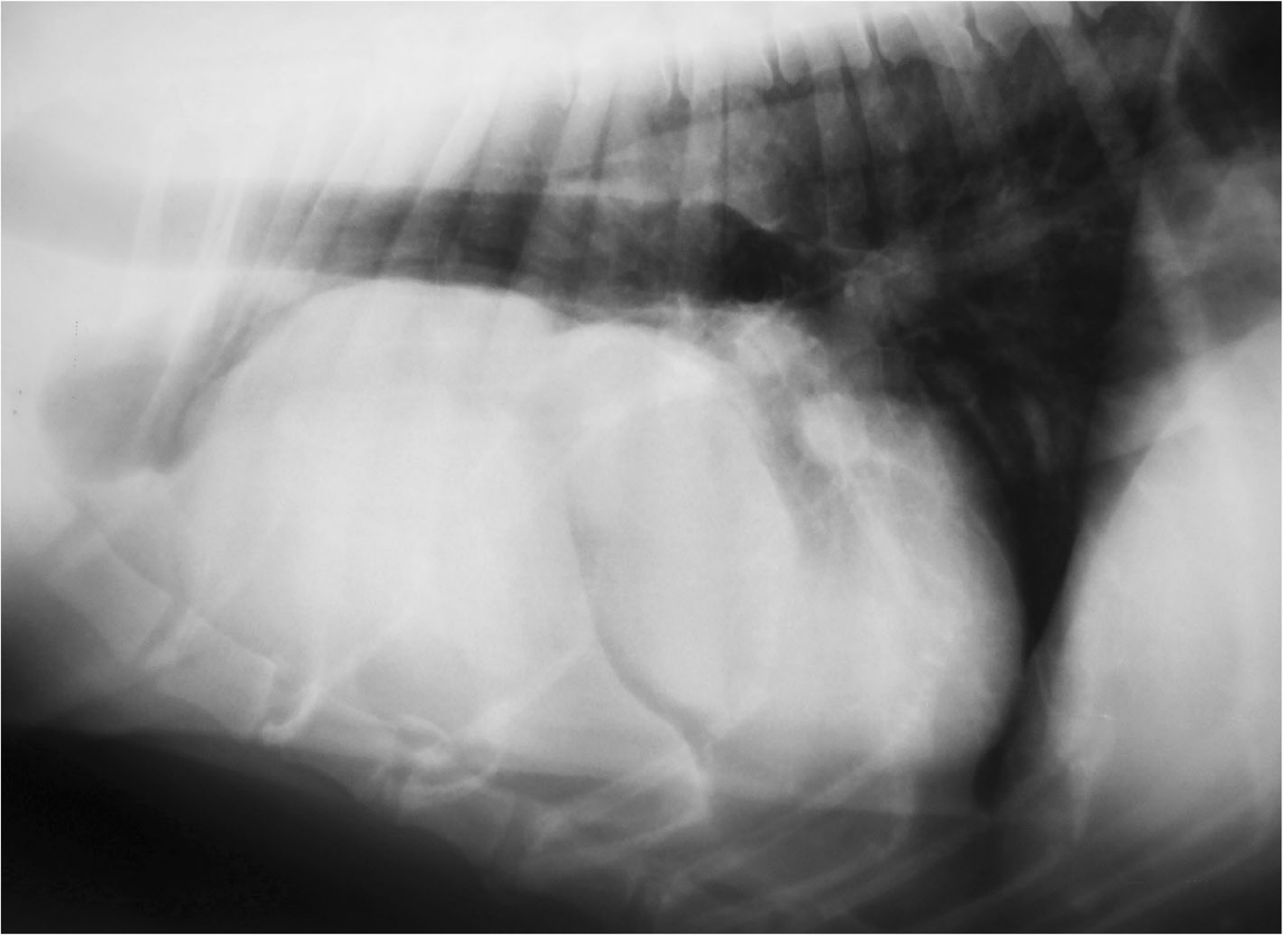
Thoracic radiograph of the dog in the laterolateral view revealed a large mass of soft-tissue density in the left cranial chest wall involving intercostal spaces 1–7 and a small round pulmonary mass in the cranial lobe of the left lung

Thoracic radiographs revealed a large mass of soft-tissue density in the left cranial chest wall involving intercostal spaces 1–7 and a small round pulmonary mass in the cranial lobe of the left lung (Fig. 1). Thoracic ultrasonography confirmed a large bilobed mass measuring at least 210 mm × 160 mm. The lesion was heterogeneous and hypoechoic with smooth borders. Mild pleural effusion was present.

Given the medical history of the dog, the main differential diagnosis was metastasis from the mammary carcinoma removed 19 months earlier. The second step was cytology of the chest wall mass, which indicated a possible MCT. The owner was informed of the diagnosis of a possible MCT, with systemic signs possibly caused by hyperhistaminemia.

After 3 days of supportive therapy, the condition of the dog improved, and the dog was adequately hydrated and began eating. An intercostal thoracoscopy revealed a thoracic wall mass involving the internal intercostal muscle that would require a radical resection and a small mass in the left cranial lobe of the lung. A thoracoscopic biopsy was obtained from both masses. No further masses were detected. A thoracostomy tube was placed under direct endoscopic visualisation, and a three-layer closure was used to close the incisions. Analgesia was achieved by fentanyl infusion (1–5 μg/kg/h) during the postoperative period, meloxicam (0.1 mg/kg, PO, once daily) and lidocaine local injection (4 mg/kg).

**Fig. 2:**
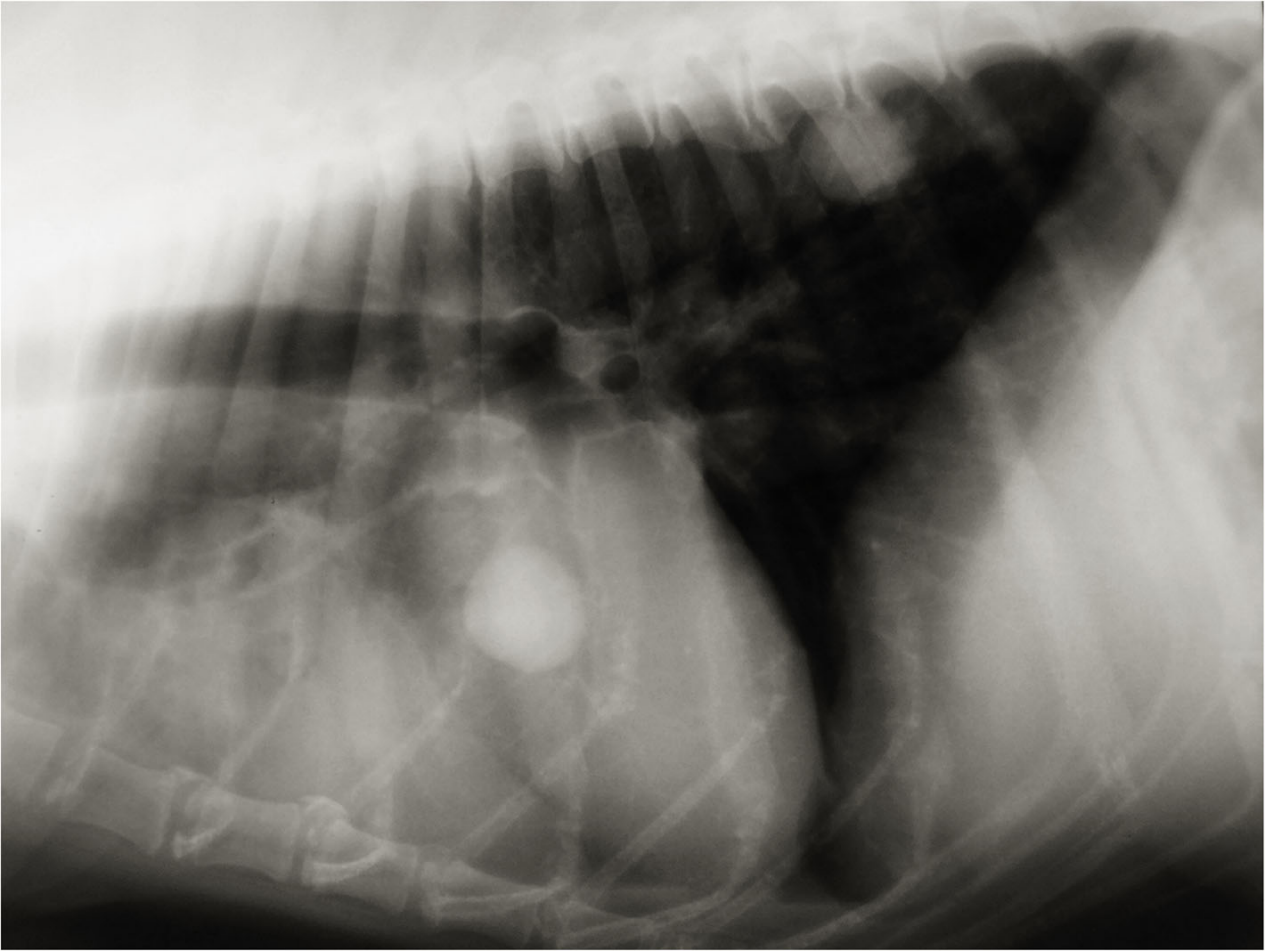
Haematoxylin-eosin staining (× 100) of the large mass biopsy sample revealed a highly cellular sample with a predominant population of discrete round cells with a pale basophilic cytoplasm and intracytoplasmic granules (purple). Each cell had central nuclei with dispersed chromatin and a single prominent nucleolus. Mild anisocytosis and anisokaryosis were present. These round cells were morphologically consistent with mast cells

**Fig. 3:**
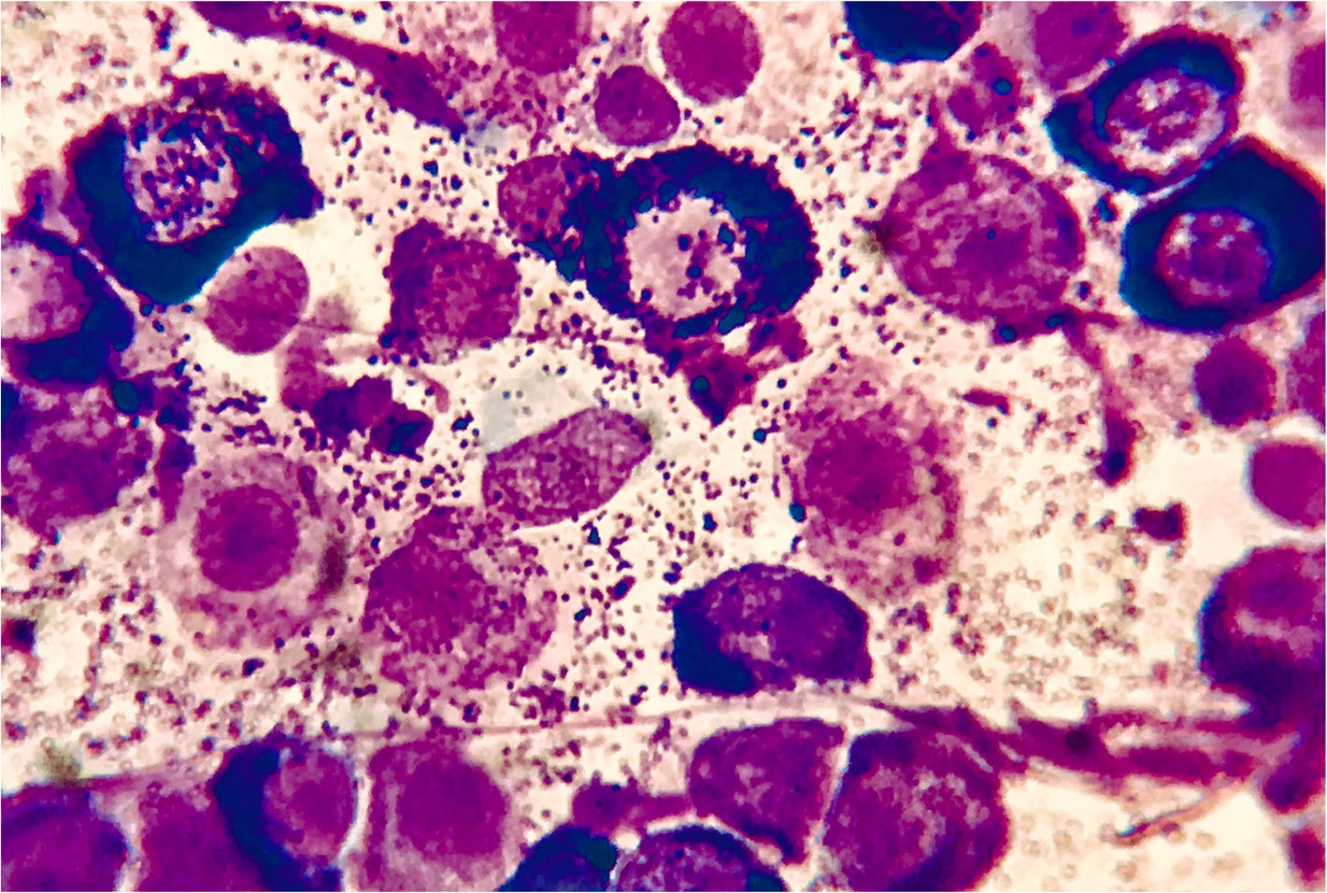
Immunohistochemical expression pattern of the KIT protein (CD117) in a primary intrathoracic chest wall mast cell tumour in a dog. The KIT protein is a type III tyrosine kinase protein involved in mast cell growth and differentiation (× 400). Courtesy of Thompson Phatology

Cytology and histopathology of the intrathoracic mass confirmed the large mass as a MCT (Fig. 2) and the small mass as a carcinoma. Cytology of the sternal lymph nodes, liver and spleen showed no involvement. Furthermore, the KIT protein staining pattern and the c-kit gene mutational status were evaluated and were positive in the large mass (Fig. 3).

**Fig. 4:**
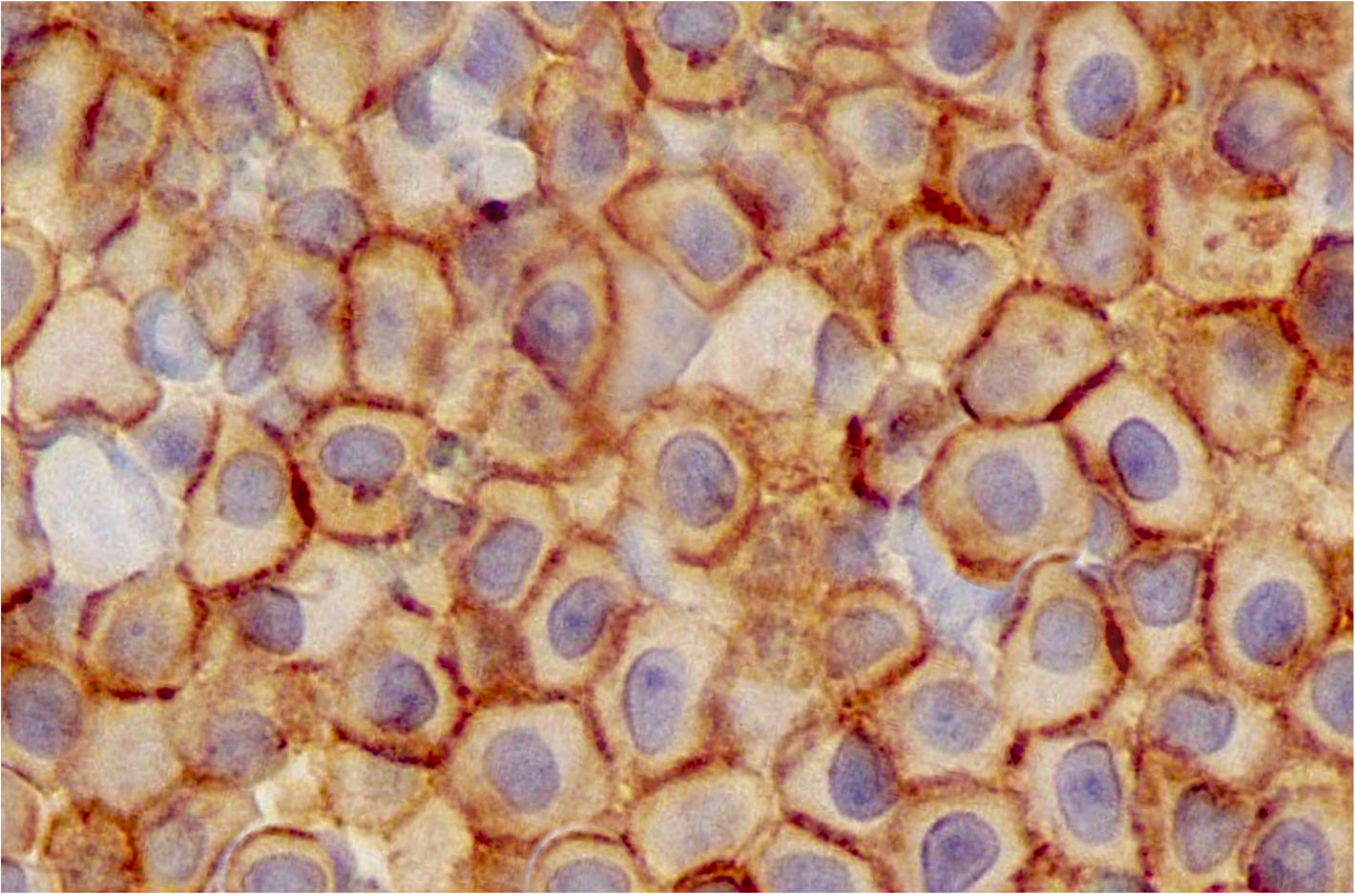
A new thoracic radiograph of the dog in the laterolateral view revealed that the large mass had a partial response but that the small mass increased in size, and a new mass in the lung, close to the spine, was visible

During hospitalisation, the dog received toceranib (2.75 mg/kg, BW, 3 times per week). After 41 days, her breathing worsened. New thoracic radiographs revealed that the large mass had shrunk, showing a partial response, but the small mass had increased in size, and a new mass was visible in the lung close to the spine (Fig. 4). No further masses were found during abdominal ultrasonography. However, based on the definitive prognosis and observed deterioration in the quality of life, the owner elected to have the dog euthanised. Post-mortem examination was not authorised by the owner.

## Case presentation 2

The medical history of the case 2 included repeated attempts to swallow solid food resulted in regurgitation. Written consent from the owner was obtained before all procedures.

A 14- year- old, 6.2- kg (body condition score 5/9), female, neutered Maltese dog presented with lethargy, weight loss, cough and dyspnoea. Upon presentation, the dog was hyperthermic (39.6 °C), apathetic and approximately 6% dehydrated. She was tachycardic (168 beats/min), a soft abdominal palpation and no palpable external masses.

Blood analysis and supportive therapy were initially performed. A complete blood cell count, serum biochemistry profiling and analyses of venous blood gases and electrolytes were performed. All results were within reference limits.

The dog received intravenous crystalloid fluid therapy and was medicated with amoxicillin and clavulanic acid and ranitidine.

**Fig. 5:**
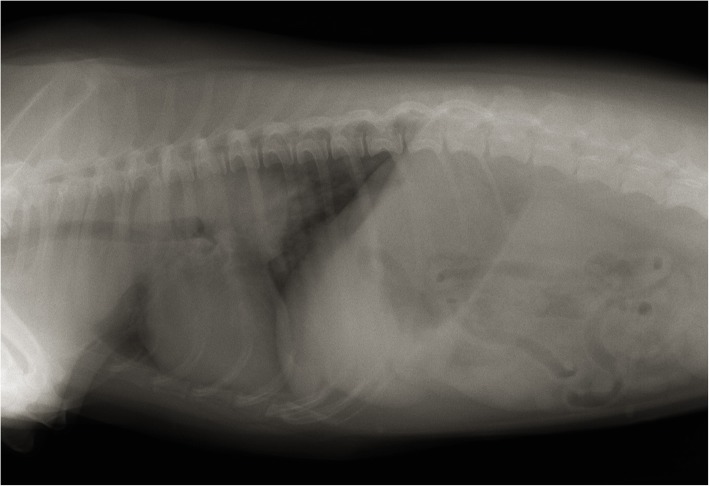
Thoracic radiographs revealed a large mass of soft-tissue density in the caudal lobe of the left lung involving intercostal spaces 5–8

**Fig. 6:**
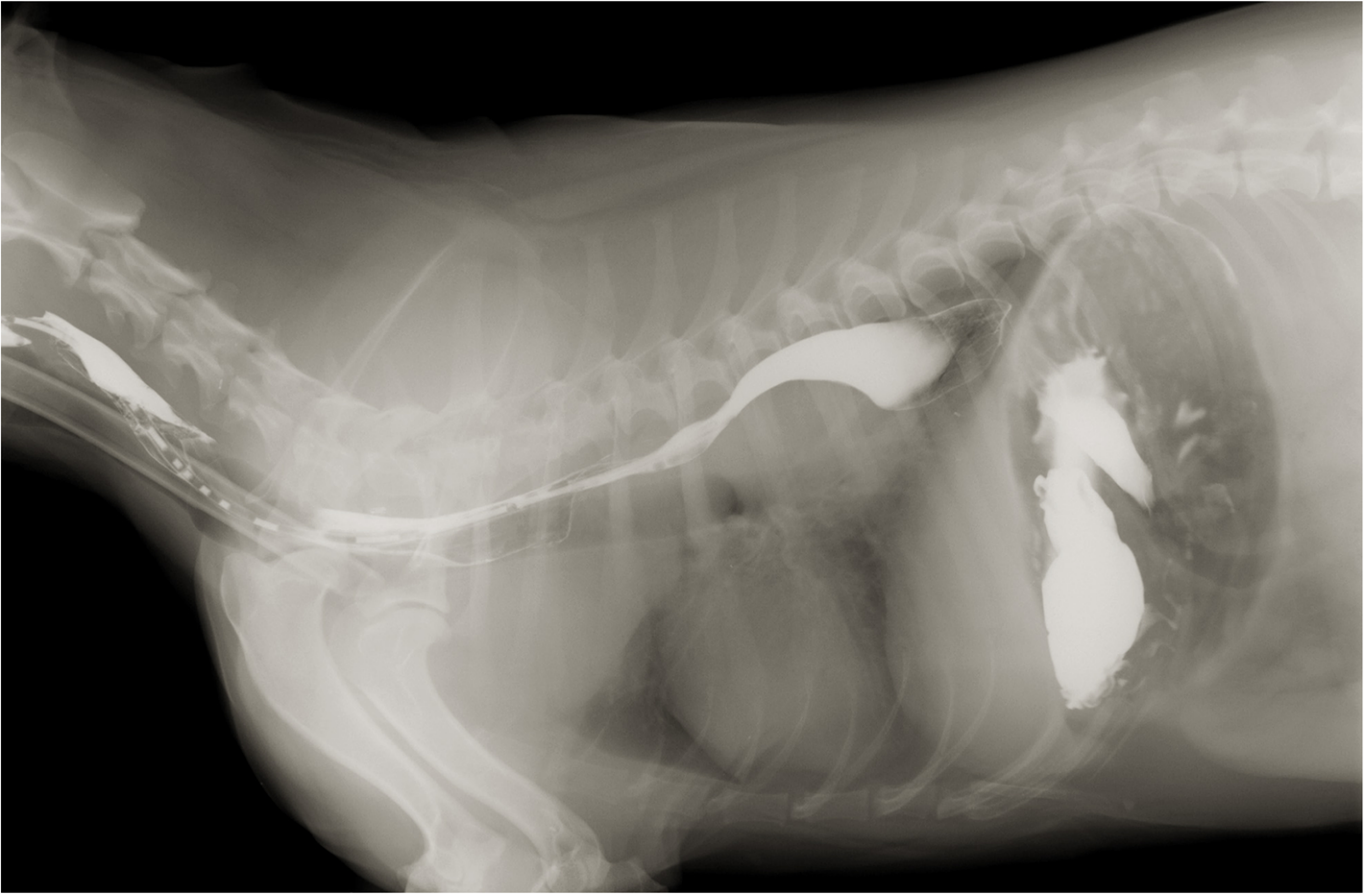
Contrast radiographies with barium sulphate demonstrated the presence of the mass and a partial blockage of esophagus

Occasional episodes of productive cough and regurgitation were observed during hospitalization. Thoracic radiographs revealed a large mass of soft-tissue density in the caudal lobe of the left lung involving intercostal spaces 5–8 (Fig. 5). Contrast radiographies with barium sulphate demonstrated the presence of the mass and the partial blockage of esophagus (Fig. 6).

**Fig. 7:**
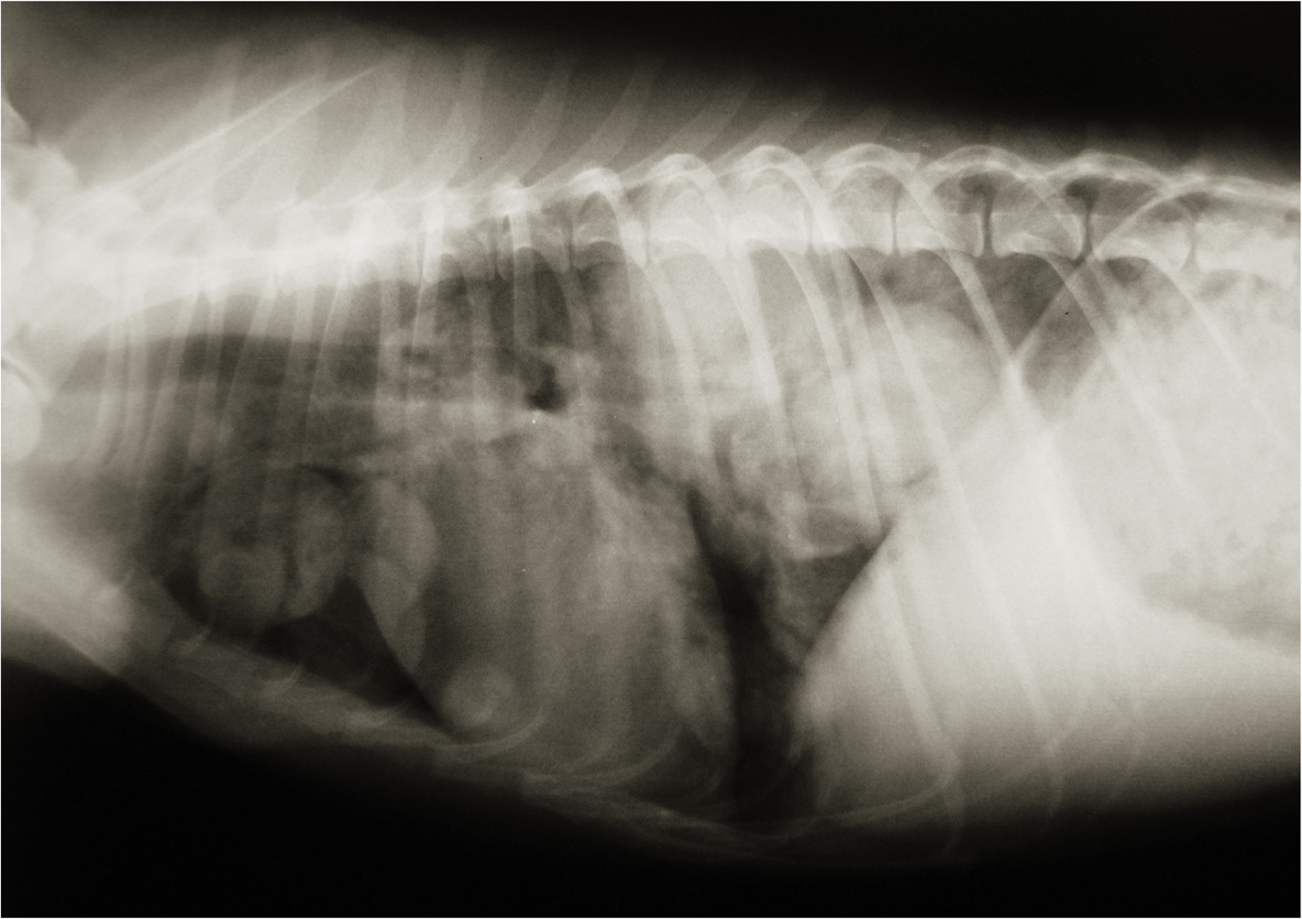
New thoracic radiographs revealed that the large mass had increased in size, and new masses were visible in the lungs and cranial medastinal lymph node. The owner elected to have the dog euthanised

**Fig. 8:**
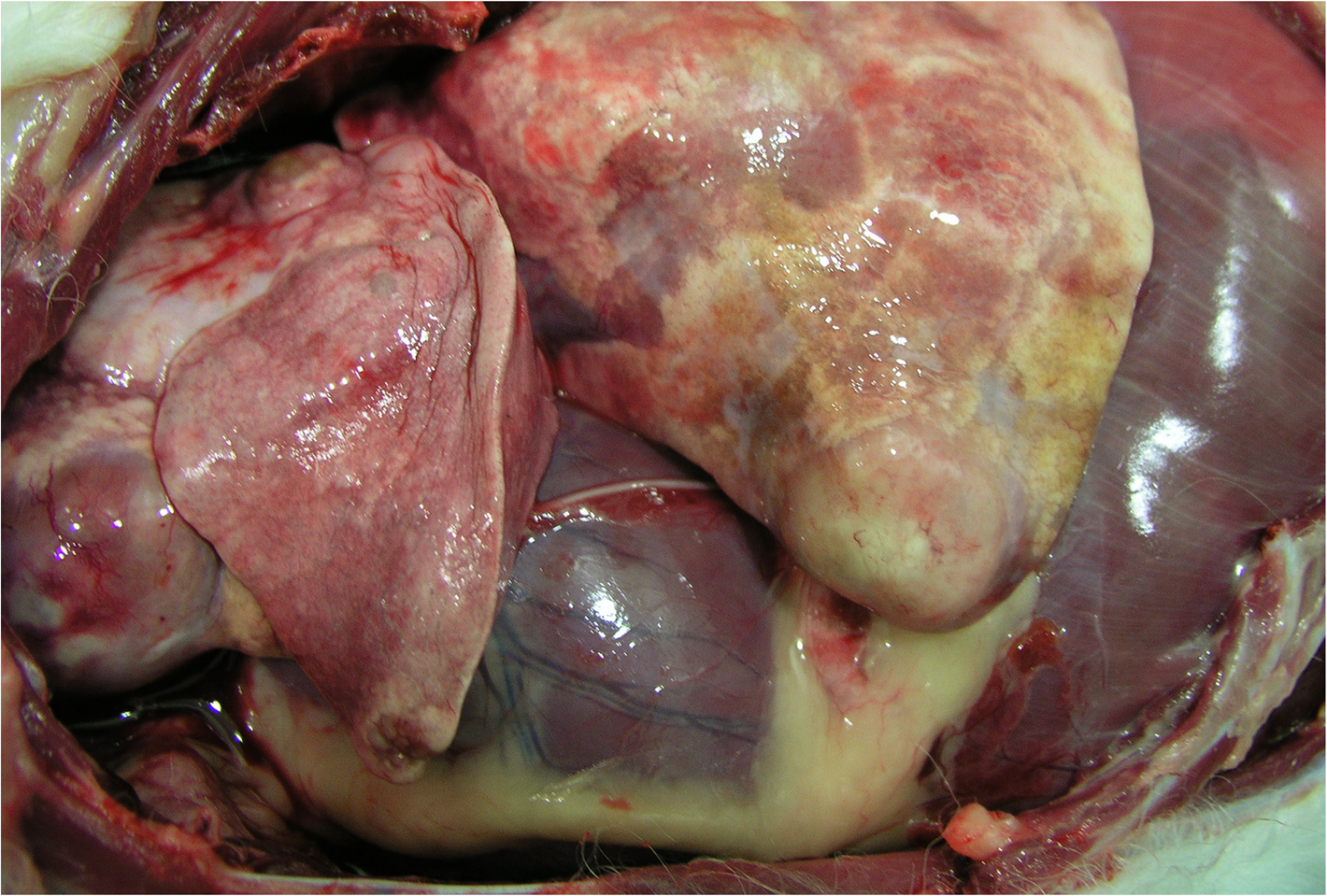
Post-mortem examination of the dog with confirmation of lung mast cell tumour and the absence of cutaneous mast cell tumour

The second step was cytology of lung mass, which indicated a possible MCT. The owner was informed of the diagnosis of a possible MCT, with systemic signs caused by the partial blockage of the esophagus. A thoracoscopic biopsy was obtained from the mass. No further masses were detected. Histopathology of the intrathoracic mass confirmed a MCT. Cytology of the liver and spleen showed no involvement. The dog received toceranib and, after 56 days, her breathing worsened. New thoracic radiographs revealed that the large mass had increased in size, and new masses were visible in the lungs and cranial medastinal lymph node (Fig. 7). The owner elected to have the dog euthanised. Post-mortem examination was authorised by the owner. Confirmation of lung MCT and the absence of cutaneous MCT was achieved by postmortem examination (Fig. 8).

## Discussion and conclusions

In this report, we described a presumptive primary intrathoracic chest wall MCT and a presumptive primary lung MCT without a previous history of cutaneous MCT in two dogs.

Cytology is very important in the diagnosis of MCT and is often more sensitive than histopathology [10]. A cytological diagnosis of MCT was obtained in the current cases, and histopathology further confirmed the diagnosis. The dog presented with a large intrathoracic chest wall MCT. Presentation of pulmonary MCT has been reported in 2 dogs without a previous history of cutaneous MCT [5], and there is also report of a case of pulmonary MCT with a concurrent splenic mass [11]. The clinical presentation of this cases study showed a different and unusual presentation of an intrathoracic MCT.

The sensitivity of cytopathological analysis has been reported to be higher than that of ultrasonography for the detection of mast cell infiltration in the spleen and liver [12]. Cytology of the spleen and liver has been performed in these cases, although the organs appeared normal on ultrasonography and showed no involvement.

The clinical staging of Case 1 showed intrathoracic mast cell disease. Thus, it can be presumed that this is the first reported case of canine primary intrathoracic chest wall MCT. A limitation of this case study was the lack of post-mortem examination, bone marrow biopsy and buffy coat smear. A post-mortem examination and a bone marrow examination could have showed additional lesions corresponding to mast cell disease at non-pulmonary sites. However, a buffy coat smear has low sensitivity and specificity for the detection of circulating malignant mast cells [13].

The clinical staging of Case 2 showed intrathoracic mast cell disease. Thus, it can be presumed that this is one of the first reported cases of canine primary lung MCT. In this case, the post-mortem examination confirmed the diagnostic. A limitation of this case was the lack of bone marrow biopsy and buffy coat smear. A bone marrow examination could have showed additional lesions corresponding to mast cell disease at non-pulmonary sites.

Furthermore, flow cytometry or lymphocyte immunochemistry markers could have been used to exclude a possible mast cell hyperplastic condition secondary to a lymphoproliferative cause. Similarly, flow cytometry and immunochemistry could have excluded round cell neoplasia, confirming the diagnosis of MCT [14]. In these cases, cytology and histopathology confirmed mast cell disease, and thus, it seems unlikely that a different diagnosis would have been obtained.

In the Case 1, the staining pattern for the KIT protein and the c-kit gene mutational status were evaluated and were positive. In the last decade, tyrosine kinase inhibitors, such as toceranib and masitinib, have been developed to treat canine MCT via the inhibition of KIT signalling. The large mass demonstrated a partial response to toceranib, but the small mass grew, and a new mass appeared in the last radiograph.

Cytoplasmic secretory granules of mast cells (histamine, heparin, proteases, etc) can lead to gastrointestinal ulceration via stimulation of hydrochloric acid production [3]. Plasma histamine concentrations are higher in dogs with MCT than in clinically healthy dogs [15]. Advanced disease may be correlated with a progressive elevation in plasma histamine concentrations [8]. Gastrointestinal signs were detected in both cases [3].

In conclusion, the dog discussed in the Case 1 presented with a large intrathoracic chest wall MCT with no evidence of either pre-existing or concurrent cutaneous MCT. The dog discussed in the Case 2 presented with a large intrathoracic lung MCT with no evidence of either pre-existing or concurrent cutaneous MCT. They were very unusual presentations of canine MCT. However, the incidence of four presumptive primary intrathoracic MCTs is not sufficient to support a statistically significant phenomenon. A well-controlled study is thus necessary to confirm that MCTs should be included in the differential diagnosis of intrathoracic tumours.

## Data Availability

‘Not applicable’.

## Abbreviation

MCT: mast cell tumour
