# Correction to: Presumptive primary intrathoracic mast cell tumours in two dogs

**DOI:** 10.1186/s12917-021-02852-7

**Published:** 2021-04-02

**Authors:** Juan Carlos Cartagena-Albertus, Antoaneta Moise, Sergio Moya-García, Nora Cámara-Fernández, Jose Alberto Montoya-Alonso

**Affiliations:** 1 Northlands Vets, 2 Northampton road, Kettering, NN15 7JU UK; 2 Vetersalud Dr. Moya, Av Joan Miró, 40, 29620 Torremolinos, Spain; 3 Internal Medicine, Faculty of Veterinary Medicine, University Institute for Biomedical and Health Research (IUIBS), University of Las Palmas de Gran Canaria, Campus Universitario Cardones de Arucas, 35413 Las Palmas de Gran Canaria, Spain

**Correction to: BMC Vet Res 15, 204 (2019)**

**https://doi.org/10.1186/s12917-019-1950-5**

In response to the publication of the article, readers brought to the author’s attention that there are some inaccuracies in the original published article [1].

1. In the abstract, it is stipulated that thoracic metastasis of mast cell tumours (MCT) has not been reported in dogs, however this is inaccurate and therefore the abstract should be amended to the following:

**Abstract**

**Background**

**Mast cell tumours are the most common cutaneous neoplasms in dogs. Other primary sites include visceral organs, such as the gastrointestinal tract, liver, or spleen, and the oral cavity. Frequent metastatic sites include the local lymph nodes, skin, spleen, liver and bone marrow. The thorax is rarely affected by metastatic disease.**

**Mast cell tumours are usually not considered as a differential diagnosis for lung and intrathoracic chest wall masses in dogs. Chest wall tumours can be primary tumours of the ribs and sternum, an invasion of adjacent tumours into the chest wall, and metastasis from distant tumours.**

**Case presentation**

**A German Shepherd dog presented with a history of persistent cough and a large mass involving the thoracic wall and a small round pulmonary mass. The dog had a history of mammary tumours that were surgically excised. Thoracoscopy revealed a thoracic wall mass involving the internal intercostal muscle and a small mass in the left cranial lung lobe. Cytology and histopathology of the intrathoracic mass confirmed the large mass as a mast cell tumour and the small mass as a carcinoma. Cytology of the sternal lymph nodes showed no involvement. The dog received toceranib for 3 months, which failed to alleviate persistent cough. Radiology indicated that the large mass had a partial response to toceranib. The dog was euthanized.**

**A Maltese dog presented with a history of chronic regurgitation and cough, and a large mass involving the left caudal lung lobe. Cytology and histopathology of mass confirmed a mast cell tumour. The dog received toceranib for 2 months. Radiology indicated that the large mass had no response to toceranib. The dog was euthanized. Confirmation of lungs mast cell tumour and the absence of any other Mast cell tumour was achieved by postmortem examination.**

**Conclusions**

**The cases discussed are two unusual presentations of intrathoracic mast cell tumours, in the absence of cutaneous mast cell tumours, in dogs.**

2. In the background section, it was stated that:

“Frequent metastatic sites for canine MCTs include the local lymph nodes, skin, spleen, liver and bone marrow. The intrathoracic chest wall or lungs are rarely affected by metastatic disease of a MCT [3], and no such cases have been reported in dogs.”

And

“We found no reference to canine primary intrathoracic MCTs. In all reported cases of intrathoracic MCT, the mast cell disease always spreads to extrathoracic organs [1, 4, 6, 7, 9]. To the best of our knowledge and based on the presentation of a large solitary intrathoracic chest wall lesion and a pulmonary mass and the absence of previous cutaneous MCT, these cases study represent the first reported instances of canine presumptive primary MCTs involving the intrathoracic chest wall and the lungs.”

However, this is not the case. As reference [11] in the published article reported, both cases included in this published study were presumed to have primary pulmonary MCT without evidence of cutaneous or abdominal involvement.

Therefore, the above quoted text should read as the following:

**“Frequent metastatic sites for canine MCTs include the local lymph nodes, skin, spleen, liver and bone marrow. The intrathoracic chest wall or lungs are rarely affected by metastatic disease of a MCT [3].”**

**“We found no reference to confirmed canine primary intrathoracic MCTs. In all reported cases of intrathoracic MCT, the mast cell disease always spreads to extrathoracic organs [1, 4, 6, 7, 9]. To the best of our knowledge and based on the presentation of a large solitary intrathoracic chest wall lesion and a pulmonary mass and the absence of previous cutaneous MCT, these cases study represent one of the first reported instances of canine presumptive primary MCTs involving the intrathoracic chest wall and the lungs.**

In light of the corrections to the background, the following corrections should also be applied to the Discussion and Conclusions. In the Discussion and Conclusions, it was stated that:

“Cytology is very important in the diagnosis of MCT and is often more sensitive than histopathology [10]. A cytological diagnosis of MCT was obtained in the current cases, and histopathology further confirmed the diagnosis. The dog presented with a large intrathoracic chest wall MCT. Presentation of pulmonary MCT has been reported in 2 dogs without a previous history of cutaneous MCT [5], and there is also report of a case of pulmonary MCT with a concurrent splenic mass [11]. The clinical presentation of this cases study showed a different and unusual presentation of an intrathoracic MCT.”

And

“The clinical staging of Case 1 showed intrathoracic mast cell disease. Thus, it can be presumed that this is the first reported case of canine primary intrathoracic chest wall MCT. A limitation of this case study was the lack of post-mortem examination, bone marrow biopsy and buffy coat smear. A post-mortem examination and a bone marrow examination could have showed additional lesions corresponding to mast cell disease at non-pulmonary sites. However, a buffy coat smear has low sensitivity and specificity for the detection of circulating malignant mast cells [13].”

This text is corrected to read as this following:

**“Cytology is very important in the diagnosis of MCT and is often more sensitive than histopathology [10]. A cytological diagnosis of MCT was obtained in the current cases, and histopathology further confirmed the diagnosis. The dog presented with a large intrathoracic chest wall MCT. Presentation of pulmonary MCT has been reported in 2 dogs without a previous history of cutaneous MCT [11], and there is also report of a case of pulmonary MCT with a concurrent splenic mass [14]. The clinical presentation of this cases study showed a different and unusual presentation of an intrathoracic MCT.”**

And

**“The clinical staging of Case 1 showed intrathoracic mast cell disease. Thus, it can be presumed that this one of the first reported case of canine primary intrathoracic chest wall MCT. A limitation of this case study was the lack of post-mortem examination, bone marrow biopsy and buffy coat smear. A post-mortem examination and a bone marrow examination could have showed additional lesions corresponding to mast cell disease at non-pulmonary sites. However, a buffy coat smear has low sensitivity and specificity for the detection of circulating malignant mast cells [13].”**

3. In the published article, the legends for the Figs. 2, 3, and 4 did not match the figures presented. The corrected figures and associated legends are presented below.

**Figure** **2****Corrected**

**Fig. 2:**
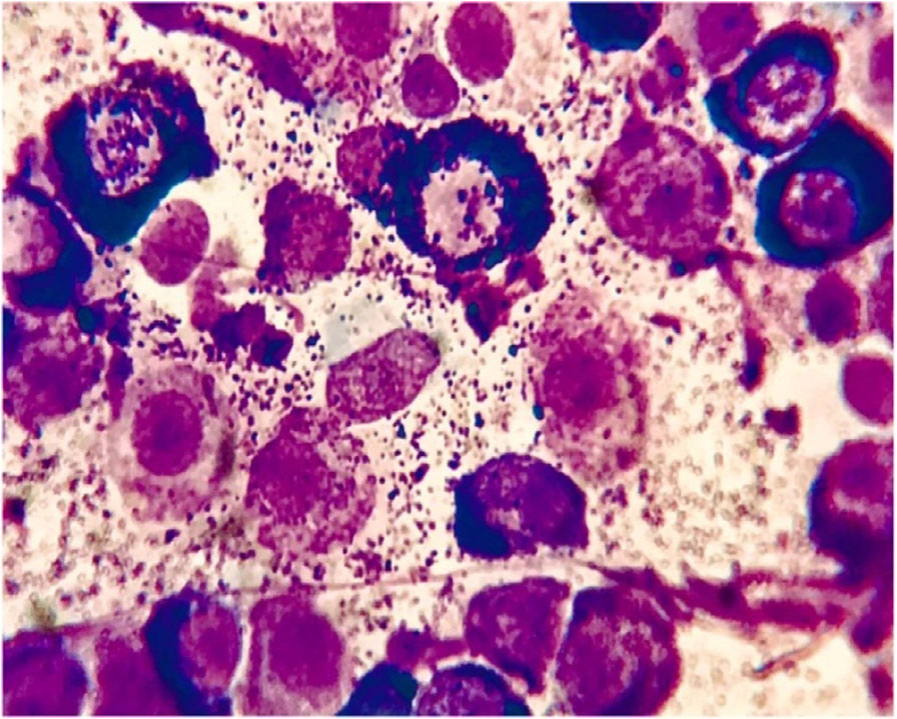
Haematoxylin-eosin staining (× 100) of the large mass biopsy sample revealed a highly cellular sample with a predominant population of discrete round cells with a pale basophilic cytoplasm and intracytoplasmic granules (purple). Each cell had central nuclei with dispersed chromatin and a single prominent nucleolus. Mild anisocytosis and anisokaryosis were present. These round cells were morphologically consistent with mast cells

**Figure** **3****Corrected**

**Fig. 3:**
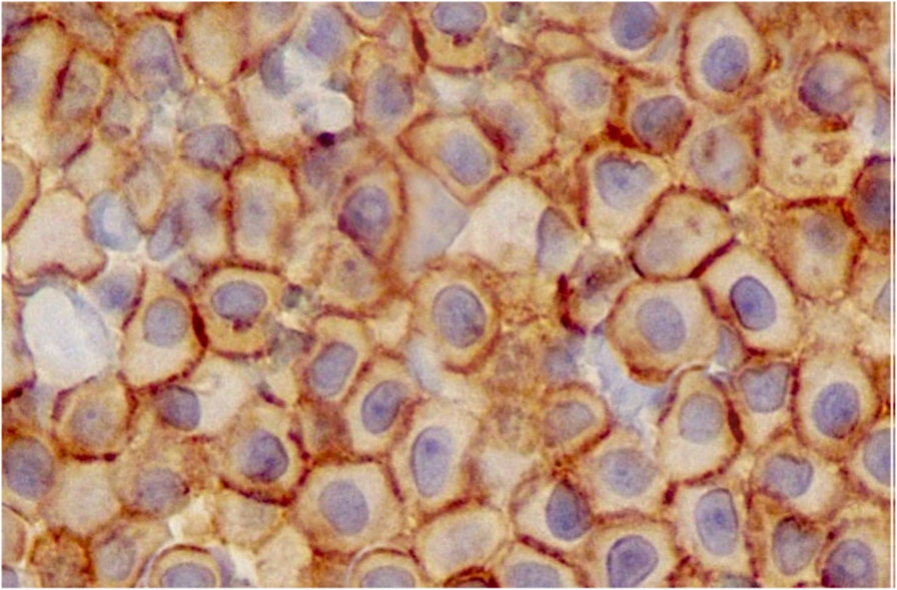
Immunohistochemical expression pattern of the KIT protein (CD117) in a primary intrathoracic chest wall mast cell tumour in a dog. The KIT protein is a type III tyrosine kinase protein involved in mast cell growth and differentiation (× 400). Courtesy of Thompson Phatology

**Figure** **4****Corrected**

**Fig. 4:**
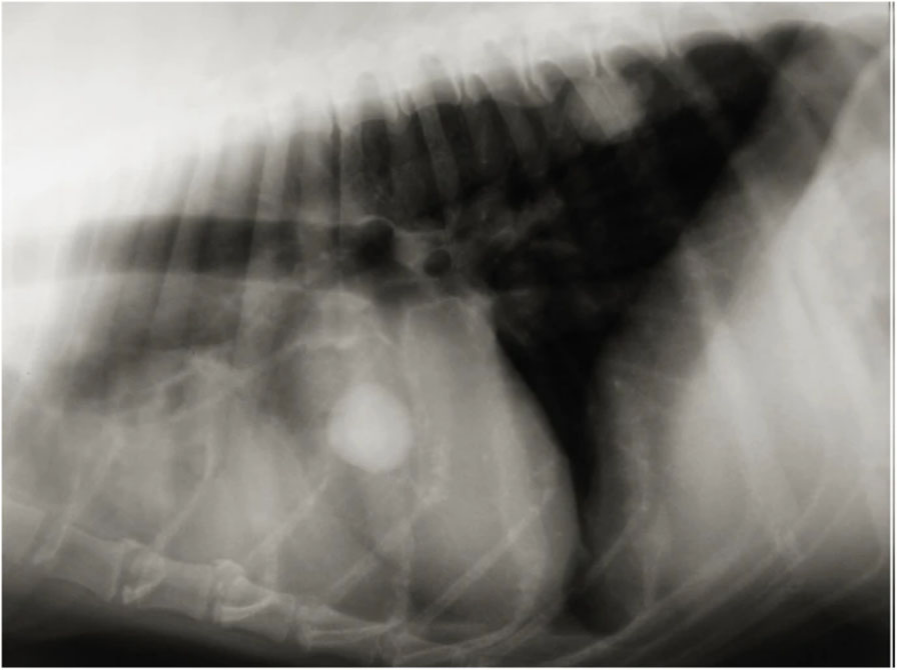
A new thoracic radiograph of the dog in the laterolateral view revealed that the large mass had a partial response but that the small mass increased in size, and a new mass in the lung, close to the spine, was visible
